# Evaluation of Housekeeping Genes for Quantitative Real-Time PCR Analysis of *Bradysia* *odoriphaga* (Diptera: Sciaridae)

**DOI:** 10.3390/ijms17071034

**Published:** 2016-07-07

**Authors:** Caihua Shi, Fengshan Yang, Xun Zhu, Erxia Du, Yuting Yang, Shaoli Wang, Qingjun Wu, Youjun Zhang

**Affiliations:** 1 College of Agriculture, Yangtze University, Jingzhou 434025, China; shicaihua1980@126.com (C.S.); nxy@yangtzeu.edu.cn (Y.Y.); 2 College of Life Science, Heilongjiang University, Harbin 150080, China; jingronghu@126.com; 3 Department of Plant Protection, Institute of Vegetables and Flowers, Chinese Academy of Agricultural Sciences, Beijing 100081, China; nxy2005@126.com (X.Z.); wangshaoli@caas.cn (S.W.); wuqingjun@caas.cn (Q.W.); 4 Department of Medicine, University of Connecticut Health Center, Farmington, CT 06030, USA; du@uchc.edu

**Keywords:** *Bradysiaodoriphaga*, normalization, reference genes, RefFinder

## Abstract

The soil insect *Bradysia* *odoriphaga* (Diptera: Sciaridae) causes substantial damage to Chinese chive. Suitable reference genes in *B. odoriphaga* (*Bradysia* *odoriphaga*) have yet to be identified for normalizing target gene expression among samples by quantitative real-time PCR (qRT-PCR). This study was focused on identifying the expression stability of 12 candidate housekeeping genes in *B. odoriphaga* under various experiment conditions. The final stability ranking of 12 housekeeping genes was obtained with RefFinder, and the most suitable number of reference genes was analyzed by GeNorm. The results revealed that the most appropriate sets of internal controls were *RPS15*, *RPL18*, and *RPS18* across developmental phases; *RPS15*, *RPL28*, and *GAPDH* across temperatures; *RPS15* and *RPL18* across pesticide treatments; *RSP5*, *RPS18*, and *SDHA* across photoperiods; *ACTb*, *RPS18*, and *RPS15* across diets; *RPS13* and *RPL28* across populations; and *RPS15*, *ACTb*, and *RPS18* across all samples. The use of the most suitable reference genes versus an arbitrarily selected reference gene resulted in significant differences in the analysis of a target gene expression. *HSP23* in *B. odoriphaga* was found to be up-regulated under low temperatures. These results will contribute to the standardization of qRT-PCR and will also be valuable for further research on gene function in *B. odoriphaga*.

## 1. Introduction

Quantitative real-time PCR (qRT-PCR) is considered as a reliable technique for the gene quantification [1,2,3]. However, gene expression can be affected by many confounding factors, such as RNA extraction, reverse transcription, and qRT-PCR efficiency [4,5]. Therefore, housekeeping genes are commonly used as “reference genes” to decrease the effects due to confounding factors and to increase the accuracy of the quantification analysis related to the particular biological environment [6,7]. The reference genes overcome the whole steps of the analyses along with interest genes and suppress the variations within the treatment group to the lowest level. Determining the number and identity of the reference genes to be employed for count data of normalization factors (NF) among comparable samples is indispensable for the precise quantification of gene expression. Thus far, however, qRT-PCR remains unreliable because of unquestioning selection of reference genes and random decision of the number for data standardization. In most of the insect samples thus far studied, for example, the expression levels of frequently used reference genes show unacceptable variability among tissues or under different physiological conditions [8,9]. The use of such reference genes will lead to inaccurate calculations and may hide true differences among samples or may indicate false differences [10]. Gutierrez et al. found that estimates of gene expression level can differ by 100-fold depending on the selection of reference gene [11]. It follows that before a housekeeping gene is applied as a reference gene, its stability should be evaluated in the particular tissue and under the particular experimental conditions of the study [12,13]. In addition, at least two or three reference genes with stable expression pattern should be selected [14,15,16].

Although qPCR has been frequently utilized for detecting expression in insects, there is still no suitable housekeeping gene and stable gene quantification system for the chive gnat, *Bradysia* *odoriphaga* Yang and Zhang (Diptera: Sciaridae). It has been reported that the chive gnat is a major soil pest of Chinese chive, *Allium tuberosum* Rottler ex Sprengel [17,18,19]. With its high fecundity, overlapping generations, and wide host range, the chive gnat occurs throughout China [20,21]. The chive gnat commonly reduces the yield of Chinese chive by 40%–60% and in some cases destroys the entire crop [22,23,24,25]. Quantitative examination of gene expression in *B. odoriphaga* (*Bradysia* *odoriphaga*) may increase our understanding of the biology and control of this pest.

This study was focused on identifying suitable housekeeping genes for assessing gene expression in *B. odoriphaga* under various experimental conditions that included differences in developmental stage, temperature, population, pesticide exposure, diet, and photoperiod. We also assessed the significance of variations by comparing different normalization strategies with the merits of using the most appropriate versus a randomly selected reference genes under different temperature treatments.

## 2. Results

### 2.1. Amplification Efficiencies

**Figure 1:**
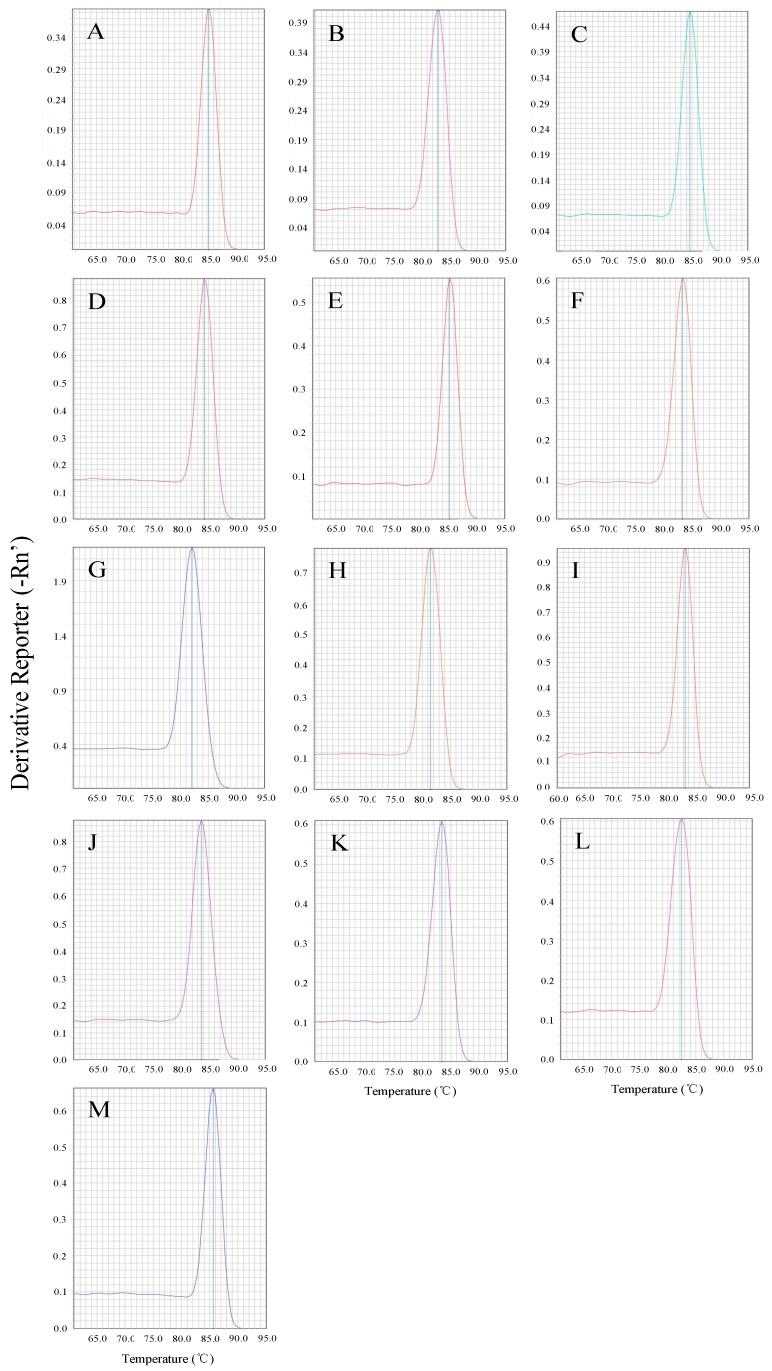
Melting curve analysis of quantitative real-time PCR (qRT-PCR) amplification (using gene-specific primers) of 12 housekeeping gene and a target gene in *B. odoriphaga*: (**A**) *ACTb*; (**B**) *EF1a*; (**C**) *GAPDH*; (**D**) *RPL18*; (**E**) *RPL28*; (**F**) *RPS15*; (**G**) *RPS18*; (**H**) *RSP5*; (**I**) *RPS13*; (**J**) *SDHA*; (**K**) *TUB*; (**L**) *UBCE*; and (**M**) *HSP23*.

Reverse transcription PCR (RT-PCR) showed that all 12 selected reference genes and one target gene were observed in the *B. odoriphaga* samples. For each gene, an amplicon of the correct size was evident. In order to estimate the amplification efficiency of the candidate genes, five-point standard curves were drawn based on the known RNA standards concentration, and the melting curve showed a single peak in each case (Figure 1). Amplification efficiencies ranged from 95.1% to 107.0%. Coefficients of determination (*R*^2^) based on linear regression were >0.990 (Table 1).

### 2.2. Expression Images of Candidate Reference Genes

**Figure 2:**
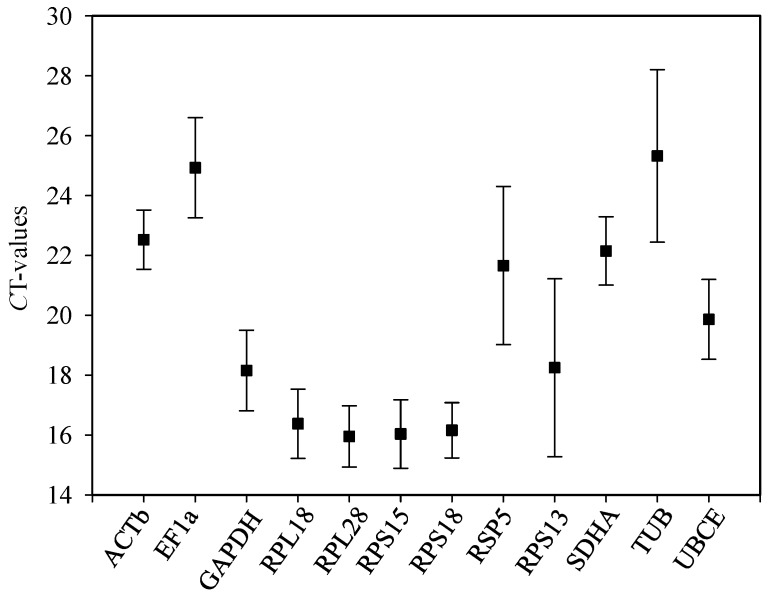
Expression profiles of the 12 housekeeping genes in all specimens of *B. odoriphaga* as indicated by cycle threshold (*C*_t_) values. Samples were from the assays with developmental stages, temperatures, populations, pesticides, diets, and photoperiods. Values are means ± SD.

To analyze mRNA expression level of the 12 candidate housekeeping genes, *C*_t_ values were calculated for all samples in this study. As shown in Figure 2, the mean *C*_t_ values of the 12 candidates were <30. The average *C*_t_ value was lowest for *RPL28* (15.95) and highest for *TUB* (25.32).

### 2.3. Stability of Reference Genes

The following results are based on analyses across the range of each factor. For developmental stage, for example, stability is based on an analysis across all stages.

#### 2.3.1. Developmental Stages

According to the four algorithms, *TUB* and *EF1a* were the least steady across developmental stage (Table 2). The most stable genes (in order) were *RPS15*, *RPL18*, and *ACTb* according to the Δ*C*_t_ method; *RPS18*, *RPS13*, and *RPL28* according to BestKeeper; *SDHA*, *ACTb*, and *GAPDH* according to NormFinder; and *RPL18*, *RPS15*, and *RPS18* according to GeNorm (Table 2).

**Figure 3:**
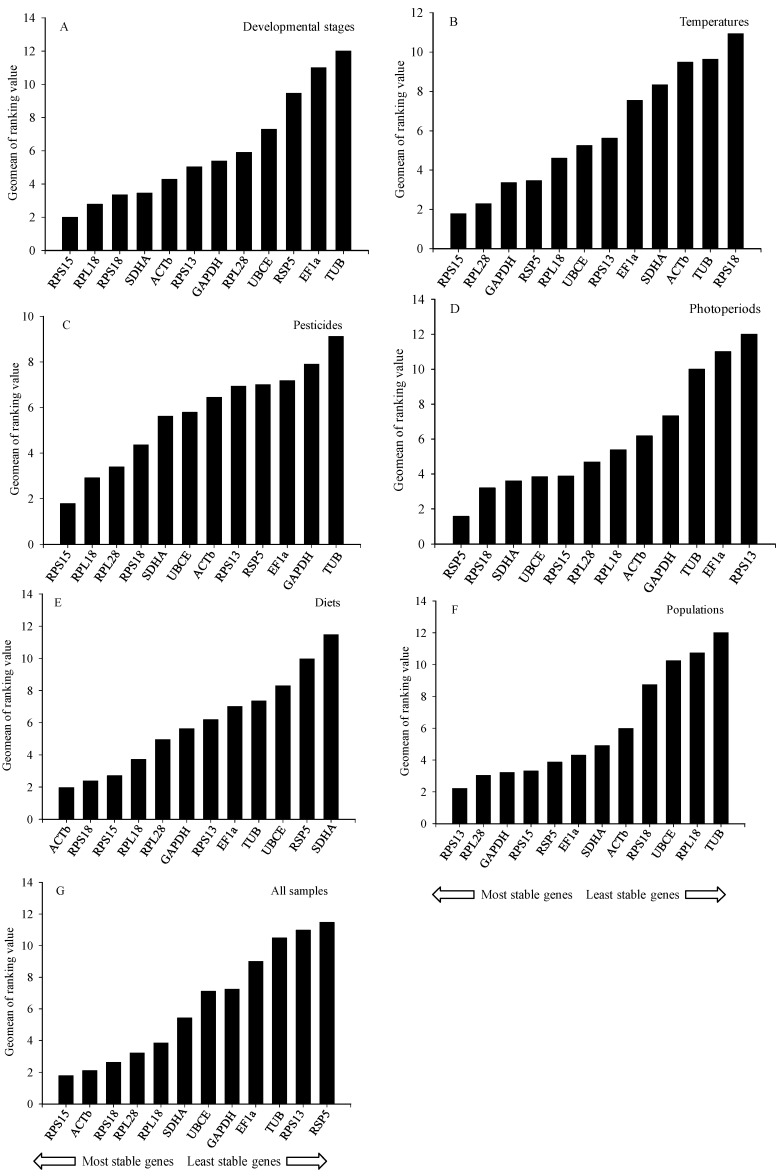
The stability of the 12 housekeeping genes in *B. odoriphaga* based on the Geomean method of RefFinder and measured across: (**A**) developmental stages (from adult to pupa); (**B**) temperatures; (**C**) pesticides; (**D**) photoperiods; (**E**) diets; (**F**) *B. odoriphaga* populations; and (**G**) all samples. For (**B**–**F**), 4th-instar larvae were used.

**Figure 4:**
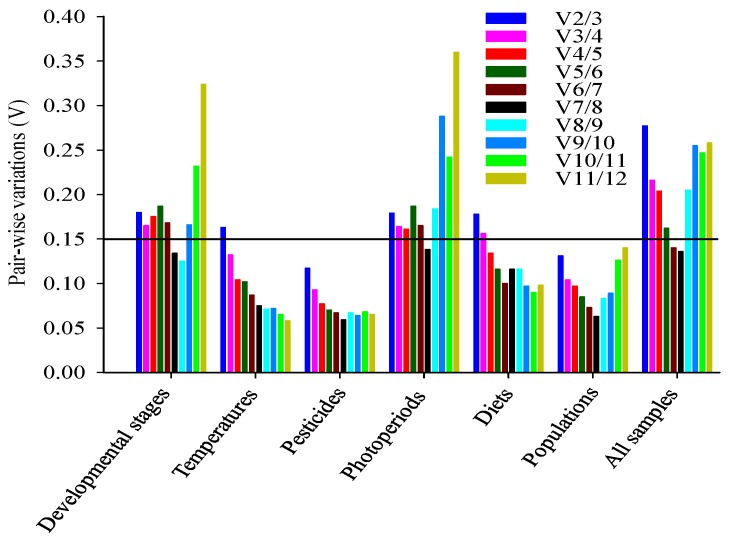
Pair-wise variation (V_n_/V_n + 1_) analysis of the number of candidate reference genes in *B. odoriphaga*. Pair-wise variation was analyzed by GeNorm software. A value <0.15 indicates that the normalization could not be dramatically changed by additional reference genes.

According to RefFinder, the order of the reference gene stability across developmental stages was: *RPS15* > *RPL18* > *RPS18* > *SDHA* > *ACTb* > *RPS13* > *GAPDH* > *RPL28* > *UBCE* > *RSP5* > *EF1a* > *TUB* (Figure 3A). GeNorm analysis results showed that the pair-wise values of V2/3 to V6/7 were all above the cut-off value of 0.15 but that the pair-wise value of V7/8 was <0.15 (Figure 4); a value <0.15 indicates that the supplemental reference genes will not evidently change the normalization. Based on the RefFinder recommendations for selection of reference genes and on convenience of operation, *RPS15*, *RPL18*, and *RPS18* were considered suitable reference genes across developmental stages of *B. odoriphaga* (Table 3).

#### 2.3.2. Temperatures

According to the Δ*C*_t_ method and NormFinder, the most steady candidate genes across temperature treatments were *RPS15*, *RPL28*, and *GAPDH*, and the least stable were *RPS18*, *SDHA*, and *TUB* (Table 2). According to BestKeeper, the most stable candidate genes were *RPL28*, *RPS15*, and *UBCE*, and the least steady were *RPS18*, *EF1a*, and *ACTb* (Table 2). According to GeNorm, the most stable candidates were *RPL18*, *RSP5*, and *RPL28*, and the least stable were *TUB*, *SDHA*, and *ACTb* (Table 2).

According to RefFinder, the order of reference gene stability across temperatures was: *RPS15* > *RPL28* > *GAPDH* > *RSP5* > *RPL18* > *UBCE* > *RPS13* > *EF1a* > *SDHA* > *ACTb* > *TUB* > *RPS18* (Figure 3B). The GeNorm data predicted that the pair-wise values from V2/3 to V3/4 were <0.15 (Figure 4). Therefore, *RPS15*, *RPL28*, and *GAPDH* were considered stable candidate genes across the tested temperatures (Table 3).

#### 2.3.3. Pesticides

*TUB*, *GAPDH*, and *EF1a* were regarded as the least steady genes across pesticide treatments by the Δ*C*_t_ method and by GeNorm and NormFinder but not by BestKeeper (Table 2). According to the comparative Δ*C*_t_ method and GeNorm, the most stable candidates were *RPS15*, *RPL18*, and *RPL28* (Table 2), while they were *RPS15*, *RPL18*, and *RPS18* by using NormFinder and were *SDHA*, *EF1a*, and *ACTb* according to BestKeeper (Table 2).

Based on RefFinder, the order of reference gene stability across pesticide treatments was: *RPS15* > *RPL18* > *RPL28* > *RPS18* > *SDHA* > *UBCE* > *ACTb* > *RPS13* > *RSP5* > *EF1a* > *GAPDH* > *TUB* (Figure 3C). The GeNorm analysis showed that the pair-wise value of V2/3 was <0.15 (Figure 4). Therefore, *RPS15* and *RPL18* were considered suitable candidate genes across the tested pesticide treatments (Table 3).

#### 2.3.4. Photoperiods

According to the four algorithms, the least stable genes across photoperiod treatments were *RPS13*, *EF1a*, and *TUB* (Table 2). The most stable genes were *RSP5*, *RPS15*, and *SDHA* according to the comparative Δ*C*_t_ method; *RSP5*, *ACTb*, and *RPS18* according to BestKeeper; *RSP5*, *SDHA*, and *RPL28* according to NormFinder; and *RPS18*, *UBCE*, and *RPL18* according to GeNorm (Table 2).

According to RefFinder, the order of reference gene stability across photoperiod treatments was: *RSP5* > *RPS18* > *SDHA* > *UBCE* > *RPS15* > *RPL28* > *RPL18* > *ACTb* > *GAPDH* > *TUB* > *EF1a* > *RPS13* (Figure 3D). The GeNorm analysis data showed that only the pair-wise value of V7/8 was below the cut-off value of 0.15 (Figure 4). *RSP5*, *RPS18*, and *SDHA* were considered to be the most stable candidate genes across photoperiod treatments (Table 3).

#### 2.3.5. Diets

Both NormFinder and Δ*C*_t_ method results shared the same stable genes (*ACTb*, *RPS18*, and *RPS15*) across diets and confirmed *SDHA*, *EF1a*, and *RSP5* as the least steady genes across diets (Table 2). According to BestKeeper, the most steady genes were *RPS15*, *EF1a*, and *GAPDH*, and the least stable were *RPL18*, *UBCE*, and *SDHA* (Table 2). According to GeNorm, the most stable genes were *RPL18*, *RPS18*, and *ACTb*, and the least stable were *SDHA*, *RSP5*, and *EF1a* (Table 2).

According to RefFinder, the ranking order of reference gene stability across diets was: *ACTb* > *RPS18* > *RPS15* > *RPL18* > *RPL28* > *GAPDH* > *RPS13* > *EF1a* > *TUB* > *UBCE* > *RSP5* > *SDHA* (Figure 3E). The GeNorm analysis showed that the pair-wise value of V4/5 was <0.15 (Figure 4). Therefore, *ACTb*, *RPS18*, and *RPS15* were considered fitted reference genes across diets (Table 3).

#### 2.3.6. Populations

Across *B. odoriphaga* populations, *TUB*, *RPL18*, and *UBCE* were identified as the least stable genes by all the four algorithms (Table 2). The most stable genes were *RPS13*, *RPS15*, and *GAPDH* according to the comparative Δ*C*_t_ method; *RPL28*, *SDHA*, and *GAPDH* according to BestKeeper; *RPS13*, *RPS15* and *RPL28* according to NormFinder; and *EF1a*, *RSP5*, and *GAPDH* according to GeNorm (Table 2).

According to RefFinder, the order of reference gene stability across populations was: *RPS13* > *RPL28* > *GAPDH* > *RPS15* > *RSP5* > *EF1a* > *SDHA* > *ACTb* > *RPS18* > *UBCE* > *RPL18* > *TUB* (Figure 3F). The GeNorm analysis showed that V2/3 value was <0.15 (Figure 4). Therefore, *RPS13* and *RPL28* were considered suitable reference genes for gene expression (Table 3).

### 2.4. Ranking of Reference Genes for All Specimens

Across all samples, the three computational programs, and the comparative Δ*C*_t_ method ranked *RSP5*, *RPS13*, and *TUB* as the least stable genes (Table 2). The most stable genes were *RPS15*, *ACTb*, and *RPL18* according to the Δ*C*_t_ method; *RPS18*, *ACTb*, and *RPL28* according to BestKeeper; *ACTb*, *RPS15*, and *RPS18* according to NormFinder; and *RPL28*, *RPS15*, and *RPL18* according to GeNorm (Table 2). Based on RefFinder, the order of reference gene stability across all samples was: *RPS15* > *ACTb* > *RPS18* > *RPL28* > *RPL18* > *SDHA* > *UBCE* > *GAPDH* > *EF1a* > *TUB* > *RPS13* > *RSP5* (Figure 3G). The GeNorm analysis showed that only the pair-wise values of V6/7 to V7/8 were less than the cut-off value of 0.15 (Figure 4). Therefore, *RPS15*, *ACTb*, and *RPS18* were regarded as the most suitable reference genes for qRT-PCR (Table 3).

### 2.5. Target Gene Expression

**Figure 5:**
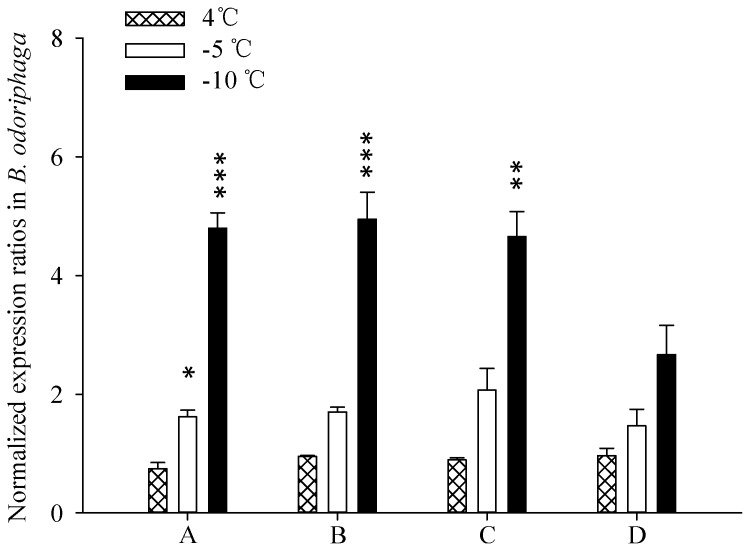
Relative expression of a target gene, *HSP23*, was affected by three temperature treatments and standardized with different numbers, and kinds of reference genes. The expression level was separately normalized by: A (*RPS15*); B (*RPS15* and *RPL28*); C (RPS15, *RPL28* and *GAPDH*); or D (*ACTb*) reference genes. The reference genes were selected depending on the expression stability of the 12 housekeeping genes among the three temperature treatments. Values are means ± SD of three biology replications; the “*” means remarkable differences, * *p* < 0.05; ** *p* < 0.01; *** *p* < 0.001.

The selection failure of internal controls led to remarkable differences in quantification target genes. The relative expression level of *HSP23* significantly differed among temperature treatments (4, −5, or −10 °C) when normalized by the most stable reference genes (such as *RPS15*) (Figure 5). Similar changes observed in analyzing relative expression level of *HSP23* with the normalization of two reference genes (such as *RPS15* and *RPL28*) (Figure 5) or three reference genes (such as *RPS15*, *RPL28*, and *GAPDH*) (Figure 5). *HSP23* in *B. odoriphaga* was found to be up-regulated under low temperatures, especially when the temperature was below −10 °C. However, *HSP23* expression did not significantly differ among these treatments when expression was calculated with an arbitrary reference gene (such as *ACTb*) (Figure 5).

## 3. Discussion

Results obtained with qRT-PCR depend on several critical factors including RNA quantity, primer efficiency, and an internal control, i.e., a reference gene. When mRNA expression level is determined by qRT-PCR, the RNA must be intact, and primer efficiency must be determined [26]. Here, the OD ratio (A_260_/A_280_) of all RNA samples were between 1.8 and 2.0, and the amplification efficiency of the 12 candidates ranged from 90% to 110% (all *R*^2^ > 0.990) (Table 1). Thus, the quality of the RNA and amplification was sufficient for qRT-PCR.

Previous researches have reported that expression level of reference genes is not always stable under all experimental conditions [27,28,29] and that mRNA expression levels varied among several housekeeping genes [2,30]. These earlier findings were confirmed in the current study with *B. odoriphaga* (Table 2). In the current study, none of the candidate genes exhibited the same level of expression under all experiment conditions [31]. This indicates that reference genes need to be optimized and chosen depending on experimental parameters. Our data showed that, among the tested genes, mRNA expression of *RPS15* was the most stable across development stages, temperatures, pesticide treatment, and all samples of *B. odoriphaga*, which is consistent with previous studies concerning development stage and temperature treatments for *Nilaparvata* *lugens* [9] and insecticide treatments for *Helicoverpa* *armigera* [32]. In *B. odoriphaga*, *RSP5* was the most stable gene across photoperiod treatments, while RPS13 was the most stable across populations.

Previous studies have reported high expression stability for genes in the ribosomal protein (RP) genes family [27,33]. For example, among different organs, geographic populations, pesticide treatments, and starvation treatments, expression stability in *Nilaparvata* *lugens* was highest for *RPS11* [9]; among different organs and developmental stages of *Tetranychus* *cinnabarinus*, expression stability was highest for *RPS18* [34]; in *Phenacoccus* *solenopsis*, expression stability among temperature treatments was highest for *RPL32* [35]; among different developmental stages of *Schistocerca* *gregaria*, expression stability was highest for *RPL49* [36]; among different organs and developmental stages of *Cimex* *lectularius*, expression stability was highest for *RPL18* [37]; in *Spodoptera* *litura*, expression stability among different larval tissues, populations, and food treatments was highest for *RPL10* [33]; in *Plutella* *xylostella*, expression stability among different developmental stages and photoperiods was highest for *RPS13* [38]; in response to virus infection in *Tribolium* *castaneum*, expression stability was highest for *RPS3* [39]; and in *Helicoverpa* *armigera*, expression stability among temperature treatments was highest for *RPL28* [40]. As a principal component of ribosomes, ribosomal protein (RP) is important in intracellular protein biosynthesis, DNA repair, cell differentiation, etc. [31]. These results indicate that ribosomal protein genes might be useful as reference genes in interest gene expression studies. In the current study with *B. odoriphaga*, however, an exception was that *RPS13* showed the least steady expression across photoperiod treatments. Another exception was reported for *Rhodnius* *prolixus*: *RPL26* was the most variable gene in the salivary glands of starved and non-starved specimens [41].

Because actin is the main structural protein of the cellular skeleton and is important for cell function [42], expression of the actin gene is substantial in most cell types [43]. The actin gene is the most stable gene in *Chilo* *suppressalis* [44], *Schistocerca gregaria* [36], and *Apis mellifera* [45]. Our study showed that *ACTb* is an ideal reference gene in *B. odoriphaga* subjected to diet treatments. In *Helicoverpa* *armigera*, however, *ACTb* exhibited the least stable expression in response to photoperiod and temperature treatments [40]. These results further confirmed that validating the stability of reference gene is very significant. The suitability of reference genes relative to both species and experimental conditions.

In addition to be affected by species and conditions [40], the ranking of reference gene stability is also affected by the tools used to perform the ranking. In the current study with *B. odoriphaga*, for example, the most stable genes across temperature treatments were *RPS15*, *RPL28*, and *GAPDH* by using NormFinder and Δ*C*_t_ method but were *RPL28*, *RPS15*, and UBCE due to BestKeeper. This difference in ranking probably results from differences in the statistical algorithms: while BestKeeper individually analyzes the stability among candidate reference genes, NormFinder and the Δ*C*_t_ method mainly think of the pair-wise variation between two candidate genes, and then confirm the stability of one of them [44,46]. Therefore, we used RefFinder software to comprehensively estimate the stability ranking of the 12 candidates. In addition, the optimal number of reference genes was confirmed by GeNorm, which calculates the pair-wise variation (V_n_/V_n + 1_) between the continuous standardization factors or NF (NF_n_ and NF_n + 1_) [14] (Figure 3). If the first V value (V2/3) is <0.15, this indicates that two reference genes are enough for reliable normalization [14]. Nevertheless, the most appropriate number of reference genes also appears arbitrary without proper statistical verification under appropriate experimental condition. Some analyses, for example, failed to achieve V_n_/_n + 1_ <0.15, but could get relatively stable expression genes across final ranking estimated by GeNorm [47]. The most suitable number of reference genes conforms to the steadiest NF feasible with a unique sample set and a unique panel of candidates [48].

Random selection of reference genes reduces the accuracy of detecting interest genes expression because such a standardization strategy will be either under-estimate or over-estimate the expression differences among specimens. Such as the expression level of *HSP23* among different temperature samples did not significantly differ using *ACTb* as internal control, but did significantly differ using other reference gene (such as *RPS15*) (Figure 5). Normalization with two or more stable reference genes may be demanded, and researchers have recommended that multiple normalization genes were used to get more credible results [49,50,51]. Vandesompele et al. [14] recommended that reliable normalization needs at least three reference genes, and the pair-wise variation analysis in GeNorm hinted the need to include more than two genes in the current study. According to the ranking of expression stability among the 12 candidates evaluated by RefFinder in this work, we selected *RPS15*, *RPL28*, and *GAPDH* to assess the target gene *HSP23* in *B. odoriphaga* under different temperatures; the results showed that *HSP23* expression was up-regulated by low temperature, which was consistent with an earlier study that used *RPS20* as reference gene [52]. In the current study, however, an arbitrarily selected reference gene (such as *ACTb*) failed to detect a significant effect of temperature on the expression profile of *HSP23*. Therefore, optimization of reference genes is critical for exact normalization of mRNA, especially for the subtle difference. To improve the accuracy of results, it is necessary to use the panel of selected housekeeping genes for any sample set.

## 4. Materials and Methods

### 4.1. Insects

*B. odoriphaga* was collected from a Chinese chive field on the Yang Town farm, ShunYi area (40°1′ N, 116°6′ E), Beijing, China. Individuals were reared for three generations with rhizomes of Chinese chive in an incubator (MLR-352H-PC) at 25 ± 1 °C, 70% ± 5% relative humidity, and 12:12 (L:D). The specimens were promptly put into liquid nitrogen for further RNA isolation, and then screened following 12 candidate genes and amplification efficiencies.

### 4.2. Factors that Could Affect the Expression of Housekeeping Genes

The effects of the following factors on candidate reference genes mRNA were measured: developmental stage, temperature, population, pesticide exposure, diet, and photoperiod. After “exposure” to each factor (as described in the following sections), the specimens were placed in liquid nitrogen and then saved in −80 °C fridge for further study. Each factor was assessed in three independent experiments.

#### 4.2.1. Developmental Stages

Each of the six developmental stages of *B. odoriphaga* was placed in an Eppendorf tube (1.5-mL) as follows: adults (10 per tube), eggs (200 per tube), 1st-instar larvae (20 per tube), 2nd-instar larvae (20 per tube), 3rd-instar larvae (6 per tube), 4th-instar larvae (4 per tube), and pupae (4 per tube). The tubes were frozen and stored.

#### 4.2.2. Temperatures

Groups of 20 4th-instar larvae were placed in a plastic Petri dish and exposed to 25, 4, −5, or −10 °C. After 4 h, they were exposed to 25 °C for another 24 h. Four living insects per group were then put in the tube (1.5-mL), frozen, and stored.

#### 4.2.3. Pesticides

Groups of 40 4th-instar larvae were sprayed in culture dishes (Ф = 60 mm) with the LC_90_ value of allyl isothiocyanate, lime nitrogen, or thiamethoxam. An additional group of 40 larvae was sprayed with distilled water. After 24 h at 25 °C, four living larvae per group were saved in a 1.5-mL plastic tube, frozen, and stored.

#### 4.2.4. Photoperiods

Groups of 20 4th-instar larvae in plastic Petri dishes were exposed to the following photoperiods (L:D): 24:0, 12:12, or 0:24. After 96 h, 12 individuals per group were stored with a 1.5-mL tube, frozen, and stored.

#### 4.2.5. Diets

Groups of four 4th-instar larvae were maintained in an incubator at 25 ± 1 °C, 70% ± 5% relative humidity, and 12:12 (L:D) and were provided with one of the following: ginger slices, garlic bulbs, Chinese chive rhizomes, onion bulbs, or artificial diet [53]. After three generations, four larvae were placed into a 1.5-mL Eppendorf tube, frozen, and stored.

#### 4.2.6. Populations

Larvae collected from three locations in China (Dezhou, Shandong; Baoding, Hebei; and Shunyi, Beijing) were reared on rhizomes of Chinese chive in an incubator at 25 ± 1 °C, 70 ± 5% relative humidity, and 12:12 (L:D). In the third generation, 12 4th-instar larvae from each population were placed in 1.5-mL micro centrifuge tubes (four larvae per tube), frozen, and stored.

### 4.3. Candidate Reference Genes

We assessed 12 “housekeeping” genes are known as reference genes selected from other insects. They were *EF1a*, *UBCE*, *RSP5*, *GAPDH*, *RPS18*, *RPL18*, *ACTb*, *SDHA*, *RPL28*, *RPS13*, *RPS15*, and *TUB* [33,34,36,40]. The sequences were obtained from our *B. odoriphaga* transcriptome data. The secondary structure of DNA template was predicted by the mfold web server [54], with the sets as follows: melting temperature for 60 °C; Na^+^ concentration for 50 mM; Mg^2+^ concentration for 3 mM; and linear DNA sequence. Other parameters were used as default. The primers used here were designed and checked by NCBI (National center for Biotechnology Information) Primer-BLAST, under the following conditions: primer GC content between 40% and 60%; primer melting temperature for 60 °C; and PCR products size of between 80 and 200 base pairs (Table 1).

### 4.4. Total RNA Abstraction and cDNA Synthesis

Total RNA was abstracted by the Trizol method. Each sample was homogenized with 1 mL of Trizol in a glass homogenizer following the manufacturer’s protocol (TIANGEN, Beijing, China). The quality and quantity of RNA were assessed with a Thermo Scientific NanoDrop 2000c UV-Vis spectrophotometer (Thermo Fisher Scientific Inc., Waltham, MA, USA). The quality of the nucleic acid sample was considered good if the OD ratio (A_260_/A_280_) was between 1.81 and 2.05. The cDNA was synthesized using the Trans*Script*^®^ (TAKARA, Japan) All-in-One First-Strand cDNA Synthesis SuperMix in a 20 µL volume, with 4 µL 5× Trans*Script*^®^ Buffer, 1 µg total RNA, and 1 µL gDNA Remover. Following the manufacturer’s instruction, the 20-µL mixture was reacted in a Bio-rad PCR machine for 15 min at 42 °C before both the Trans*Script*^®^ RT and gDNA remover were inactivated for 5 s at 85 °C. The cDNA was stored at −20 °C.

### 4.5. qRT-PCR

Each reaction was operated in a 20-µL solution including 0.4 µL cDNA, 10 µL 5× *TransStart*^®^ SuperMix, 0.4 µL forward primer, 0.4 µL reverse primer, and 0.4 µL 50× Passive Reference Dye. The amplification conditions for the qRT-PCR were set as follows: 94 °C for 30 s, followed by 40 cycles of 94 °C for 5 s, 60 °C for 15 s, and 72 °C for 34 s. Then, the 10-fold dilution series of cDNA was used for a standard curve. The melting curve analysis from 80 to 90 °C was used for assuring specificity of the amplified product [55]. The corresponding qRT-PCR efficiencies (E) were counted by means of the equation: E = (10^[−1/slope]^ − 1) × 100 [30,55].

### 4.6. Constancy of Gene Expression

The constancy of candidate genes was estimated by the Δ*C*_t_ method [46] and with the following software: BestKeeper [56], GeNorm [14], and NormFinder [4]. The lower the value estimated by these algorithms, the greater the stability of expression. RefFinder [57], a useful web-based tool, was applied to estimate and screen the most suitable reference genes by combining the results of the four algorithms. Based on rankings from each algorithm, RefFinder assigned a suitable weight to each gene and counted the geometric mean of the overall ultimate ranking.

### 4.7. Evaluation of a Target Gene Expression

To select the suitable reference genes from 12 candidates, we estimated latent up- or down-regulation of the *HSP23* gene in *B. odoriphaga* under different temperature treatments. Gene expression ratios were calculated by using the formula (2^−ΔΔ*C*^^t^) [58].

Δ*C*_t_ =*C*_t_ (target gene) − *C*_t_ (reference gene)

ΔΔ*C*_t_ =Δ*C*_t_ (sample) − Δ*C*_t_ (control)

### 4.8. Statistical Analysis

Results are showed as means ± SD. The means were calculated with Tukey’s test at *p* < 0.05 by the software SPSS 19.0 for Windows (SPSS Inc., Chicago, IL, USA).

## 5. Conclusions

In summary, we first systematically evaluated 12 candidate reference genes in *B. odoriphaga* under various conditions. Four algorithms (NormFinder, BestKeeper, GeNorm, and the comparative Δ*C*_t_ method) were used for evaluating the suitable reference genes. RefFinder, which was applied to combine the results of the different algorithms, then indicated that the most suitable reference genes were *RPS15*, *RPL18*, and *RPS18* across developmental phases; *RPS15*, *RPL28*, and *GAPDH* across temperatures; *RPS15* and *RPL18* across pesticide treatments; *RSP5*, *RPS18*, and *SDHA* across photoperiods; *ACTb*, *RPS18*, and *RPS15* across diets; *RPS13* and *RPL28* across populations; and *RPS15*, *ACTb*, and *RPS18* across all samples. The use of the best reference genes vs. an arbitrarily selected reference gene resulted in substantial differences in the estimation of expression of a target gene. The results of this study will be valuable for research concerning gene function in *B. odoriphaga*.

## Figures and Tables

**Table 1:** Features of the 12 housekeeping genes and one target gene in *B. odoriphaga* (*Bradysia* *odoriphaga*) *s*amples.

Gene Symbol	Gene Name	Forward Primer (5′→3′)	Reverse Primer (5′→3′)	Product Length (bp)	Efficiency (%)	*R*^2^ *
*ACTb*	β-actin	CGCCCCCGAAGAAATTGTTG	GTCACGACCGGCAATGTCTA	128	107.01	1.000
*EF1a*	Elongation factor 1 alpha	TGCAACTGCACTGCGAAAAG	ACACTTTGCCCTACCGTCTG	153	102.23	0.991
*GAPDH*	Glyceraldehyde-3-phosphate	GCTAGTGCCGGTGCTGAATA	GACGCCACAGACGAACATTG	144	100.20	1.000
*RPL18*	Ribosomal protein L18	CCAACTGGCAAGGGAACTCT	AGCTACGTCTGCGACCTCTA	160	101.26	0.998
*RPL28*	Ribosomal protein L28	CGTGCCCGACATTTTCATCA	GACCAAGCCACTGTAACGGA	180	105.18	1.000
*RPS15*	Ribosomal protein S15	ATCGTGGCGTCGATTTGGAT	CTCATTTGGTGGGGCTTCCT	164	101.03	0.997
*RPS18*	Ribosomal protein S18	AACGAGCTGGTGAATGTACCG	TGGACGACGTCAATTGTGTG	144	101.84	0.999
*RSP5*	Similar to ubiquity family member	TCTACCAAAGGCGCACACAT	CAACCGCAAATCCACACGTT	116	103.85	1.000
*RPS13*	Ribosomal protein S13	AAGTACGTTTCGTCAGCGGT	GTTTGCGAATAGCGACAGCC	117	97.35	0.999
*SDHA*	Succinate dehydrogenase	TTGCCTGCTGAACAATTGGC	GTCGGTACGCCACCCATATT	134	95.10	0.998
*TUB*	Alpha tubulin	ACAGTGCAAGGGCTTACAGG	GCTGTTGATACTCTGGGCGA	159	101.80	1.000
*UBCE*	Ubiquitin-conjugating enzyme	ACTACGGGCCGATTTAGCTG	CATTTGGTCGCTTCTCGCTG	101	102.58	0.998
*HSP23*	Small heat shock protein	GAGAGCTATGCATCGCGACA	GCATTCTGCGGGTCGATTTC	140	106.86	0.997

The gene source was transcriptome data in all cases. * Regression coefficient obtained according to standard regression curve.

**Table 2:** Expression stability of the 12 candidate housekeeping genes in *B. odoriphaga* under various experimental conditions.

Experimental Condition	Rank	Δ*C*_t_		BestKeeper		NormFinder		GeNorm	
		Gene Name	Standard Value	Gene Name	Standard Value	Gene Name	Standard Value	Gene Name	Standard Value
Developmental stages	1	*RPS15*	1.460	*RPS18*	0.559	*SDHA*	0.455	*RPL18/RPS15*	0.429
	2	*RPL18*	1.510	*RPS13*	0.628	*ACTb*	0.481		
	3	*ACTb*	1.520	*RPL28*	0.742	*GAPDH*	0.729	*RPS18*	0.530
	4	*SDHA*	1.530	*RPS15*	0.745	*RPS15*	0.757	*RPS13*	0.626
	5	*GAPDH*	1.620	*RPL18*	0.757	*UBCE*	0.810	*RPL28*	0.756
	6	*RPS18*	1.620	*SDHA*	0.824	*RPL18*	0.927	*SDHA*	0.908
	7	*UBCE*	1.640	*GAPDH*	0.856	*RPS18*	1.140	*ACTb*	1.020
	8	*RPS13*	1.710	*ACTb*	0.970	*RSP5*	1.221	*GAPDH*	1.080
	9	*RPL28*	1.770	*UBCE*	1.238	*RPL28*	1.264	*UBCE*	1.130
	10	*RSP5*	1.950	*RSP5*	1.652	*RPS13*	1.273	*RSP5*	1.259
	11	*EF1a*	2.860	*EF1a*	1.754	*EF1a*	2.514	*EF1a*	1.520
	12	*TUB*	3.990	*TUB*	3.942	*TUB*	3.870	*TUB*	1.931
Temperatures	1	*RPS15*	0.640	*RPL28*	0.298	*RPS15*	0.307	*RPL18/RSP5*	0.476
	2	*GAPDH*	0.680	*RPS15*	0.432	*GAPDH*	0.397		
	3	*RPL28*	0.690	*UBCE*	0.457	*RPL28*	0.415	*RPL28*	0.521
	4	*RSP5*	0.720	*SDHA*	0.457	*RSP5*	0.478	*GAPDH*	0.564
	5	*RPS13*	0.750	*RPS13*	0.468	*RPS13*	0.515	*RPS15*	0.581
	6	*UBCE*	0.760	*TUB*	0.486	*UBCE*	0.522	*EF1a*	0.625
	7	*EF1a*	0.770	*RPL18*	0.498	*EF1a*	0.544	*UBCE*	0.654
	8	*RPL18*	0.800	*GAPDH*	0.564	*RPL18*	0.612	*RPS13*	0.674
	9	*ACTb*	0.830	*RSP5*	0.585	*ACTb*	0.645	*RPS18*	0.696
	10	*SDHA*	0.850	*ACTb*	0.608	*TUB*	0.682	*ACTb*	0.726
	11	*RPS18*	0.860	*EF1a*	0.712	*SDHA*	0.683	*SDHA*	0.748
	12	*TUB*	0.860	*RPS18*	0.721	*RPS18*	0.695	*TUB*	0.767
Pesticides	1	*RPS15*	0.550	*SDHA*	0.277	*RPS15*	0.297	*RPL28/RPS15*	0.300
	2	*RPL18*	0.580	*EF1a*	0.305	*RPL18*	0.323		
	3	*RPL28*	0.580	*ACTb*	0.402	*RPS18*	0.356	*RPL18*	0.351
	4	*RPS18*	0.600	*TUB*	0.496	*RPL28*	0.373	*GAPDH*	0.387
	5	*UBCE*	0.610	*RPS18*	0.506	*UBCE*	0.385	*UBCE*	0.413
	6	*RPS13*	0.620	*RPL18*	0.511	*RPS13*	0.387	*RPS18*	0.438
	7	*RSP5*	0.630	*RSP5*	0.518	*RSP5*	0.424	*RSP5*	0.470
	8	*ACTb*	0.670	*RPS13*	0.585	*ACTb*	0.471	*RPS13*	0.492
	9	*GAPDH*	0.670	*UBCE*	0.632	*GAPDH*	0.536	*ACTb*	0.535
	10	*SDHA*	0.750	*RPS15*	0.656	*SDHA*	0.591	*SDHA*	0.575
	11	*EF1a*	0.830	*RPL28*	0.684	*EF1a*	0.704	*EF1a*	0.622
	12	*TUB*	0.880	*GAPDH*	0.774	*TUB*	0.765	*TUB*	0.664
Photoperiods	1	*RSP5*	1.620	*RSP5*	0.526	*RSP5*	0.324	*RPS18/UBCE*	0.542
	2	*RPS15*	1.680	*ACTb*	0.700	*SDHA*	0.363		
	3	*SDHA*	1.720	*RPS18*	0.967	*RPL28*	0.442	*RPL18*	0.580
	4	*RPL28*	1.740	*SDHA*	0.998	*RPS15*	0.523	*RPS15*	0.655
	5	*RPL18*	1.760	*RPL28*	1.035	*RPS18*	0.849	*GAPDH*	0.746
	6	*UBCE*	1.770	*UBCE*	1.047	*UBCE*	0.850	*RSP5*	0.903
	7	*RPS18*	1.780	*RPS15*	1.212	*RPL18*	0.899	*SDHA*	1.009
	8	*GAPDH*	1.900	*RPL18*	1.335	*GAPDH*	1.071	*RPL28*	1.074
	9	*ACTb*	2.040	*GAPDH*	1.592	*ACTb*	1.337	*ACTb*	1.225
	10	*TUB*	3.040	*TUB*	1.874	*TUB*	2.899	*TUB*	1.564
	11	*EF1a*	3.090	*EF1a*	2.075	*EF1a*	2.956	*EF1a*	1.778
	12	*RPS13*	4.370	*RPS13*	4.172	*RPS13*	4.300	*RPS13*	2.210
Diets	1	*ACTb*	0.850	*RPS15*	0.596	*ACTb*	0.333	*RPL18/RPS18*	0.470
	2	*RPS18*	0.860	*EF1a*	0.604	*RPS18*	0.435		
	3	*RPS15*	0.920	*GAPDH*	0.638	*RPS15*	0.550	*ACTb*	0.546
	4	*RPL18*	0.960	*TUB*	0.665	*RPL18*	0.621	*RPL28*	0.613
	5	*RPL28*	0.980	*ACTb*	0.777	*RPL28*	0.683	*RPS13*	0.673
	6	*RPS13*	1.020	*RPL28*	0.803	*GAPDH*	0.728	*RPS15*	0.719
	7	*GAPDH*	1.050	*RPS13*	0.805	*RPS13*	0.735	*UBCE*	0.752
	8	*UBCE*	1.060	*RPS18*	0.864	*UBCE*	0.801	*GAPDH*	0.825
	9	*TUB*	1.130	*RSP5*	0.928	*TUB*	0.870	*TUB*	0.900
	10	*RSP5*	1.160	*SDHA*	0.956	*RSP5*	0.920	*EF1a*	0.945
	11	*EF1a*	1.190	*UBCE*	0.980	*EF1a*	0.977	*RSP5*	0.984
	12	*SDHA*	1.340	*RPL18*	1.056	*SDHA*	1.154	*SDHA*	1.042
Populations	1	*RPS13*	0.760	*RPL28*	0.200	*RPS13*	0.189	*EF1a/RSP5*	0.405
	2	*RPS15*	0.770	*SDHA*	0.214	*RPS15*	0.247		
	3	*GAPDH*	0.770	*GAPDH*	0.366	*RPL28*	0.324	*GAPDH*	0.430
	4	*RPL28*	0.790	*RPS13*	0.404	*GAPDH*	0.364	*ACTb*	0.457
	5	*RSP5*	0.810	*ACTb*	0.406	*RSP5*	0.445	*RPS15*	0.498
	6	*SDHA*	0.830	*RPS15*	0.473	*SDHA*	0.448	*RPS13*	0.527
	7	*EF1a*	0.860	*EF1a*	0.474	*EF1a*	0.525	*RPL28*	0.551
	8	*ACTb*	0.860	*RPS18*	0.503	*ACTb*	0.546	*SDHA*	0.567
	9	*RPS18*	0.960	*RSP5*	0.517	*RPS18*	0.604	*RPS18*	0.625
	10	*UBCE*	1.080	*RPL18*	0.834	*UBCE*	0.830	*UBCE*	0.694
	11	*RPL18*	1.550	*UBCE*	0.937	*RPL18*	1.472	*RPL18*	0.829
	12	*TUB*	1.740	*TUB*	1.576	*TUB*	1.674	*TUB*	0.981
All samples	1	*RPS15*	1.630	*RPS18*	0.744	*ACTb*	0.565	*RPL28/RPS15*	0.893
	2	*ACTb*	1.650	*ACTb*	0.811	*RPS15*	0.668		
	3	*RPL18*	1.660	*RPL28*	0.828	*RPS18*	0.763	*RPL18*	0.926
	4	*RPS18*	1.670	*SDHA*	0.917	*RPL18*	0.768	*RPS18*	0.968
	5	*RPL28*	1.710	*RPS15*	0.925	*UBCE*	0.810	*ACTb*	1.054
	6	*SDHA*	1.730	*RPL18*	1.039	*SDHA*	0.826	*SDHA*	1.095
	7	*GAPDH*	1.740	*GAPDH*	1.057	*RPL28*	0.848	*GAPDH*	1.127
	8	*UBCE*	1.760	*UBCE*	1.069	*GAPDH*	0.868	*UBCE*	1.171
	9	*EF1a*	2.330	*EF1a*	1.192	*EF1a*	1.116	*EF1a*	1.354
	10	*TUB*	2.990	*RSP5*	2.125	*TUB*	2.623	*RPS13*	1.620
	11	*RPS13*	3.030	*TUB*	2.210	*RPS13*	2.774	*TUB*	1.857
	12	*RSP5*	3.320	*RPS13*	2.274	*RSP5*	3.062	*RSP5*	2.101

**Table 3:** Recommended reference genes in *B. odoriphaga* under various experimental conditions.

Experimental Condition	Reference Genes		
Developmental stages	*RPS15*	*RPL18*	*RPS18*
Temperatures	*RPS15*	*RPL28*	*GAPDH*
Pesticides	*RPS15*	*RPL18*	
Photoperiods	*RSP5*	*RPS18*	*SDHA*
Diets	*ACTb*	*RPS18*	*RPS15*
Populations	*RPS13*	*RPL28*	
All samples	*RPS15*	*ACTb*	*RPS18*
